# Paralysis in Cows and Hæmoglobinuria in Horses from the Same Cause

**Published:** 1882-07

**Authors:** 


PARALYSIS IN COWS AND HÆMOGLOBINURIA IN HORSES FROM THE SAME CAUSE.—Some interesting cases have been reported by Mayer (*Reportorium Thierheilkunde*) showing that the same cause can act in curiously different ways upon different animals. They also illustrate the necessity of veterinary hygiene.

Within six weeks four horses and six cows, kept in the same stable, were attacked—the first with hæmoglobinuria (bloody urine), the others with paralysis of the pharynx.

The history is as follows: Three horses were first attacked with bloody urine; two of them died within twenty-four hours. Then two cows became afflicted with paralysis of the pharynx. These, not being able to swallow, were, after two or three days, killed. Ten days later the remaining four cows were attacked in the same way and had to be killed. The stalls were cleaned, and two more horses placed in them. In three days they were attacked with bloody urine.

The cause was proved not to be in the food. It was concluded that it must have been in the very bad hygienic condition of the stalls. These were low, damp, badly lighted, and undrained.

